# Metacognition in adult ADHD: subjective and objective perspectives on self-awareness of cognitive functioning

**DOI:** 10.1007/s00702-020-02293-w

**Published:** 2021-01-19

**Authors:** Marah Butzbach, Anselm B. M. Fuermaier, Steffen Aschenbrenner, Matthias Weisbrod, Lara Tucha, Oliver Tucha

**Affiliations:** 1 Department of Clinical and Developmental Neuropsychology, Faculty of Behavioural and Social Sciences, University of Groningen, Grote Kruisstraat 2/1, Groningen, 9712 TS Groningen The Netherlands; 2 Department of Psychiatry and Psychotherapy, SRH Clinic Karlsbad-Langensteinbach, Karlsbad-Langensteinbach, Germany; 3 Department of Clinical Psychology and Neuropsychology, SRH Clinic Karlsbad-Langensteinbach, Karlsbad-Langensteinbach, Germany; 4 Department of General Psychiatry, Center of Psychosocial Medicine, University of Heidelberg, Heidelberg, Germany; 5 Department of Psychiatry and Psychotherapy, University Medical Center Rostock, Rostock, Germany

**Keywords:** Adult ADHD, Metacognition, Self-awareness, Neuropsychological performance

## Abstract

Although attention deficit hyperactivity disorder (ADHD) in adulthood is associated with marked cognitive impairment, research on metacognition in adult ADHD is scarce. Deficits in metacognition may have a negative impact on treatment adherence, functional outcomes, and everyday life. This study explores metacognition, specifically self-awareness of cognitive performance, in adults with ADHD by combining objective and subjective assessments. Forty-seven patients with ADHD and 47 control individuals completed a neuropsychological assessment battery including tests for attention, executive functions and memory (objective assessment), as well as questionnaires for cognitive functioning and symptom severity (subjective assessment; self- and informant-report). Participants evaluated their test performance of the objective assessment after test completion by selecting a percentile rank which was subtracted from their normed test result, yielding a discrepancy score. Compared to controls, adults with ADHD showed impairments in attention (medium effects) and memory (small and medium effects), but not in executive functions. The discrepancy scores between self-evaluation and cognitive performance revealed deficits in self-awareness of attentional functions (small effects), but not in executive functions and memory in patients with ADHD compared to controls. Discrepancy scores between self- and informant-reports of cognitive functioning revealed no significant differences**.** Adults with ADHD show impairments in metacognition in attentional functions, but may have intact metacognitive abilities in other domains. Patients with ADHD tend to overestimate their abilities, especially in attentional functions. Subjective and objective measures of metacognition may not correspond, highlighting the need for clinicians to not solely rely on patients’ self-report in their assessment.

**Supplementary Information:**

The online version contains supplementary material available at (10.1007/s00702-020-02293-w).

## Introduction

ADHD is a neurodevelopmental disorder which affects about 2.5% of adults (Simon et al. 2009). ADHD in adulthood is associated with a substantial burden, as adults with ADHD commonly report diminished functional outcomes, such as lower educational and occupational achievement, struggles in their social environment, and traffic violations (Agarwal et al. 2012; Barkley and Murphy 2010). Adult ADHD is characterized by impairments in several cognitive domains, such as attention, memory, and executive functions (Schoechlin and Engel 2005). Impairments in various aspects of attention have consistently been found, for example in selective attention and vigilance (Egeland et al. 2009; Mueller et al. 2017; Tucha L. et al. 2009; Tucha O. et al. 2006). Memory deficits have also been replicated (Fuermaier et al. 2015; Johnson et al. 2001; Schoechlin and Engel 2005), and in executive functions, a mixed picture emerged, as only 30–50% of patients may experience deficits in executive functions (Barkley and Murphy 2010; Johnson et al. 2001; Schoechlin and Engel 2005).

An aspect related to executive functioning that has hardly been investigated in adult ADHD is metacognition. Colloquially, metacognition has been defined as “thinking about thinking” or “knowing about knowing”, referring to the understanding and regulation of our own cognition (Krueger et al. 2011). Metacognition encompasses various cognitive processes, such as self-awareness, self-monitoring, and self-regulation (Knouse et al. 2005), which impact adaptive behavior in various environments (Eslinger et al. 2005). Self-awareness is essential for cognitive functioning as it allows us to select activities that are in line with our capabilities and recognize our limits (Williamson et al. 2010). For example with driving, it is easy to image how dangerous a lack of self-awareness may be (i.e., driving on the highway at night unaware of how tired one is and falling asleep on the steering wheel). Metacognition was found to influence coping, functional outcomes, and treatment adherence in patients with dementia (Williamson et al. 2010) and self-awareness of executive functioning could even predict functional decline in patients with mild cognitive impairment (Scherling 2016). In various clinical populations, deficits in self-awareness of cognitive ability were shown to have a negative impact on everyday life (Rothlind et al. 2017; Torres et al. 2016), suggesting that metacognition may be particularly relevant in the clinical context.

Yet, research on this topic is complicated by the inherent conundrum in studying metacognition: a deficit in metacognition may be hard to detect if people are asked to evaluate themselves, as they would not be able to do so realistically if they indeed have a deficit in metacognition (Nelson and Narens 1994). Therefore, self-report may not be a valid tool for investigating self-awareness. Another strategy is asking a close relative to evaluate the participant’s functioning. However, this is prone to a similar set of biases as the relative’s evaluation will also be subjective (Williamson et al. 2010). Rosen and colleagues (2014) used a postdiction discrepancy method to study metacognition in various clinical conditions, such as frontotemporal dementia and patients with HIV (Chiao et al. 2013; Rosen et al. 2010,2014; Scherling 2016; Williamson et al. 2010). In this approach, the participant’s subjective evaluation of his or her cognitive performance on a specific neuropsychological test is compared to his or her actual performance in the respective test. This self-evaluation is done after performing the respective test (postdiction). The advantage of this approach is that participant’s subjective self-assessment is compared to an (arguably) objective outside criterion. Interestingly, a deficit in self-awareness (measured by the postdiction discrepancy method) has been associated with reduced volume in the ventromedial prefrontal cortex in patients with neurodegenerative disease (Rosen et al. 2010). This is noteworthy as the ventromedial prefrontal cortex was discussed as a biomarker for ADHD (Albaugh et al. 2017), underlining the relevance of investigating metacognitive functions in ADHD.

There are several indications of deficits in self-awareness in adults with ADHD. For example, adults with ADHD reported more severe cognitive impairment on self-reports than what was found using performance tests (Fuermaier et al. 2015). Possible explanations for this discrepancy include limited ecological validity of the neuropsychological tests, a qualitative difference between the two approaches, and impaired metacognitive abilities of patients with ADHD (Fuermaier et al. 2015). Although several studies reported deficits in self-awareness and self-reflection in adults with ADHD, these findings are based on self-reports (Manor et al. 2012; Moerstedt et al. 2015) or on comparing symptom ratings of patients with ADHD with ratings made by an informant (e.g., partner or relative, Barkley et al. 2011; Jiang and Johnston 2012; Prevatt et al. 2012). Based on self-reports, the positive illusory bias (an overly positive evaluation of one’s competence that is out of line with one’s actual competence) has been well documented in children with ADHD, but received less attention in adults with ADHD (Owens et al. 2007; Prevatt et al. 2012). Overestimation of ADHD symptoms were found dependent on source of information (self or informant), which may be due to impaired self-awareness (Barkley et al. 2011), positive illusory bias of the patient (Barkley et al. 2011; Jiang and Johnston 2012; Knouse et al. 2005; Prevatt et al. 2012), but also due to factors impacting the informants’ accuracy, such as informants’ closeness to the patient and informants’ cognitive abilities (Williamson et al. 2010).

Due to the subjective nature of self- and informant-reports, there is a need for research on metacognition in adult ADHD that does not solely rely on self- and informant-reports. Yet, only a few studies applied a methodology directly connecting patients’ with ADHD subjective evaluation of their cognitive functioning with their cognitive performance (Knouse et al. 2005,2006). Knouse (2006) investigated meta-memory in adult ADHD and found that adults with ADHD did not differ from controls in their accuracy of judgement of learning, implying that in the right circumstances, they may be as good as typically developing individuals in judging their memory performance. Another study investigated self-awareness of driving in adult ADHD and found overestimations of driving abilities based on self-reports but not based on simulator performance in adults with ADHD compared to healthy controls (Knouse et al. 2005). It is essential to have a common metric between the self-evaluation and outcome measures, because otherwise the measurement accuracy may be questioned (Williamson et al. 2010). This is an advantage of the postdiction discrepancy method as participants evaluate their performance relative to others of their age based on percentiles of a normal distribution, which allows for a direct comparison of their self-evaluation with their test performance based on a normative sample.

The present study aims to explore metacognition in adult ADHD by employing the postdiction discrepancy method, comparing participants’ self-evaluation to an (arguably) objective outside criterion. Previous research on metacognition in other clinical populations found that awareness may vary across domains (Duke et al. 2002; Scherling 2016) and that metacognitive deficits may rely on different neural circuits, dependent on which type of cognitive functioning is affected (Nelson and Narens 1994). Therefore, the present study will investigate several cognitive domains which are commonly affected in adult ADHD (attention, executive functions, and memory), to elucidate whether there are differences in metacognitive functioning across domains. Given these points, adults with ADHD may differ from control participants in their metacognitive skills. Based on previous research indicating that adults with ADHD may overestimate their driving abilities (Knouse et al. 2006), we predict adults with ADHD to be less aware of their own cognitive deficits and therefore have a tendency to overestimate their cognitive performance relative to controls. Furthermore, we expect a difference in self-awareness between cognitive domains for patients with ADHD relative to controls. As almost all research on metacognitive skills in adults with ADHD so far has relied on self- and informant accounts, we aim to explore whether any difference in metacognition between patients and controls as measured by the postdiction discrepancy method can also be found by subjective accounts (self-report and informant-report) of cognitive functioning. As previous research indicated substantial discordance between patients’ subjective evaluation of their cognitive performance and their neuropsychological test performance (Barkley et al. 2011; Fuermaier et al. 2015), we do not expect the information yielded from the two sources to converge.

## Method

### Participants

#### Patients with ADHD

A total of 54 patients with ADHD participated in the study. Patients were either referred by neurologists, other professionals or self-referred to the SRH Clinic Karlsbad-Langensteinbach, Germany. Each patient underwent a structured clinical interview based on DSM 5 criteria (American Psychiatric Association 2013) conducted by clinicians of the Department of Neuropsychology at the SRH clinic. In this interview, the clinician established a history of ADHD symptomatology in childhood as well as the pr^2005^esence of current symptoms. In addition, to quantify the extent of current and past symptoms, all participants completed the Wender Utah Rating Scale (WURS-K; Ward et al. 1993), as well as the ADHD Self-Rating Scale (ASRSAdler et al. 2006; Kessler et al. ). The WURS-K assesses childhood ADHD symptomatology and includes 25 items on a 5-point Likert scale with a sum score of 30 or higher supporting the presence of ADHD symptoms in childhood. The ASRS focusses on current ADHD symptoms based on DSM diagnostic criteria (American Psychiatric Association 1994; Rösler et al. 2008) and includes 18 items on a 4-point Likert scale with a sum score of 18 or more indicating the presence of current ADHD symptoms. Furthermore, the diagnostic evaluation included objective measures such as evidence derived from school reports and reports of failure in academic and/or occupational achievement, and comprised multiple informants when possible (e.g., employer evaluation, partner, or parent reports). Patients were included based on (a) willingness to participate in the study, (b) fulfilling the diagnostic criteria of adult ADHD, and (c) being older than 18 years of age. Exclusion criteria were (a) neurological disorder including head injury, (b) schizophrenia or acute psychosis, (c) estimated verbal IQ below 85, and (d) noncredible symptom reporting and performance as indicated by two established measures of symptom and performance validity (i.e., Groningen Effort Test, Fuermaier et al. 2016, Infrequency Index of the Conners Adult ADHD Rating Scale, Conners et al. 1998; Suhr et al. 2011). Patients receiving medical treatment for ADHD (e.g., stimulants; *N* = 5) and/or depression (*N* = 12) were not excluded, as these medications are very common amongst the ADHD patient population (Zhang et al. 2020) and excluding such patients may render the sample less representative of ADHD patients as a whole. Patients receiving medications for ADHD symptomatology were asked not to take their medication on the assessment day. In applying the exclusion criteria, patients were excluded due to suspected dementia (*N* = 1), suspected psychosis with lithium and quetiapine treatment (*N* = 1), and noncredible symptom reporting and performance (*N* = 3). Furthermore, two patients were excluded as they were in acute emotional distress and the testing situation had to be interrupted. Characteristics of the remaining participants are presented in Table 1 (Appendix). Of these 47 patients with ADHD, 20 met the DSM 5 diagnostic criteria of the predominantly inattentive symptom presentation, one met the criteria of the hyperactive-impulsive presentation, and 26 met the criteria of the combined presentation. Concerning comorbidities, 35 patients with ADHD included in the sample were diagnosed with one or more psychiatric conditions, including mood disorders (*N* = 25), anxiety disorders (*N* = 15), substance dependency (*N* = 4), eating disorders (*N* = 2), personality disorders (*N* = 2), migraine (*N* = 2), autism spectrum disorder (*N* = 1), psychosomatic complaints (*N* = 1), restless legs syndrome (*N* = 1), childhood epilepsy (*N* = 1), obsessive compulsive disorder (*N* = 1), oppositional defiant disorder (*N* = 1), selective mutism (*N* = 1), and essential tremor (*N* = 1). The prevalence of comorbidities in this patient sample is roughly in line with comorbidity prevalence data in other clinical adult ADHD samples (Chen et al. 2018; Cumyn et al. 2009; Kessler et al. 2006; please see limitation section of the discussion).

#### Healthy individuals

One hundred and thirteen healthy individuals were recruited based on a community sample, such as through word of mouth and contacts of the researchers involved. Participants were included based on willingness to participate in the study and being older than 18 years. Exclusion criteria were (a) neurological disorder including head injury, (b) psychiatric or psychological disorders, (c) past diagnosis of ADHD and/or scoring above the cut-offs on both self-report scales assessing current (ADHD self-rating scale) and retrospective ADHD symptoms (Wender Utah Rating Scale), (d) medications affecting the central nervous system, (e) estimated verbal IQ below 85, and (f) noncredible symptom reporting and performance indicated by two established measures of symptom (CAARS Infrequency Index) and performance validity (GET). Consequently, 19 participants were excluded, due to ADHD symptoms (*N* = 11), depression and anxiety (*N* = 3), anxiety (*N* = 1), borderline personality disorder (*N* = 1), substance dependency (*N* = 1), obsessive compulsive disorder (*N* = 1), and noncredible symptom reporting and performance (*N* = 1). To match the two groups, for each of the 47 patients with ADHD, a control participant roughly comparable in demographic characteristics was selected. This selection was conducted on a case-by-case basis, by identifying for each patient a control participant that was as similar as possible in age, gender, IQ, and educational level.

After matching, the two groups did not significantly differ in age (*t*(92) = 0.415, *p* = 0.679), gender (*χ*^2^(1) = 1.54, *p* = 0.215), IQ (*t*(92) =  − 0.663, *p* = 0.509), and educational level (*t*(92) = 1.514, *p* = 0.133). Supporting the diagnostic status, patients with ADHD scored significantly higher in current (*t*(91) = 12.04, *p* < 0.001) and childhood symptomatology (*t*(91) = 9.60, *p* < 0.001), depressive symptomatology (*t*(90) = 7.04, *p* < 0.001), and cognitive impairment as rated by participants themselves (cognitive deficits questionnaire, self-report version, *t*(90) = 10.41, *p* < 0.001) and their relatives (cognitive deficit questionnaire, informant-report version, *t*(82) = 8.48, *p* < 0.001).

## Materials

This study was part of a larger research project including a more extensive assessment battery. This is the first study of this project. In the following, only questionnaires and neuropsychological tests relevant for the current study are described.

### Questionnaires

#### Anamnesis

A short self-made questionnaire was used to record basic demographic information and participants’ medical history. Participants were asked to indicate their age, gender, highest educational level achieved, occupation, history of medical and psychological disorders, as well as any medical treatment.

#### Beck depression inventory

Depressive symptomatology was measured with the Beck’s Depression Inventory (BDI, Beck et al. 1988). The BDI includes 21 items assessing symptoms and attitudes characterizing depression. Items are rated based on severity of the symptom and range from 0 to 3, with statements indicating varying intensity. A total score is computed by summing up the responses to all items.

#### Conner’s adult ADHD rating scale

(CAARS, Conners et al. 1998) The long version of the CAARS was used to assess adult ADHD symptomatology based on the DSM IV criteria for ADHD and to screen for noncredible responding (Suhr et al. 2011). The CAARS includes 66 items to be answered on a 4-point Likert scale (0 = not at all/never; 1 = just a little/once in a while; 2 = pretty much/often; 3 = very much/very frequently), which can be combined into eight subscales. Of interest for the present study was the Conners Infrequency Index (CII; Suhr et al. 2011), which is composed of items only very infrequently endorsed by genuine patients. A score of 21 or higher may indicate that the participant may not respond in a credible manner.

*3.1.4. Complaints of cognitive disturbances (FLei).* Self-reported cognitive deficits were assessed with the FLei (FLei is an abbreviation of the German title, which translates to “complaints of cognitive disturbances” (Beblo et al. 2010). This questionnaire asks participants to indicate to which extend examples of everyday manifestations of cognitive deficits apply to them. The FLei includes 35 items scored on a 5-point Likert scale ranging from 0 (never) to 4 (very frequently). The questionnaire is divided in 4 subscales measuring attention, executive functioning, and memory. A sum score is computed by adding the scores of the attention, executive function and memory subscales. Both a self-report and an informant-report version of this questionnaire were used. The informant-report version is identical to the self-report version with the only difference being that self-referential personal pronouns (“I”, “my” etc.) were replaced by third person pronouns (“she/he”, “her/him”, etc.).

### Neuropsychological tests

#### Verbal intelligence estimate (vocabulary skills)

Verbal intelligence was estimated with the *Multiple Choice Vocabulary Test* (MWT-B, Lehrl 1995). In this short, 37- item test for vocabulary skills participants were asked to select a real word which was intermixed with 4 made-up words. Participants receives one point for each correctly selected item, and by summing up these points, a total score is computed, which is compared to a normative sample and thereby transformed into an *IQ score*.

#### Selective attention

The Perception and Attention Functions: Selective Attention Test (WAFS) of the Vienna Test System (Schuhfried 2010; Sturm 2017a) was used to assess selective attention, which refers to the ability to assign attentional resources to a target and to inhibit reactions to distracting stimuli. In this test, circles, squares, or triangles were presented (1500 ms) consecutively on a computer screen. In some cases, the figure changed to a lighter or darker shade of gray; in most cases, the figure remained in the same shade. This change in shade represented the target stimulus upon which participants had to press a button as fast as possible. Importantly, when a triangle changed shade, participants were asked not to react. The duration of this task is 5 min. *Mean reaction time*, the *standard deviation* of reaction time, *omission,* and *commission* errors were registered.

#### Vigilance

To assess vigilance, the Perception and Attention Functions: Vigilance Test of the VTS (Schuhfried 2010; Sturm 2017b) was administered. In this vigilance task, participants need to be alert over a prolonged period of time and respond to a very infrequently occurring target. During this 15-min test, participants are shown squares at the center of a computer screen. Squares are presented consecutively for 1500 ms each and the interstimulus interval is 500 ms. The target stimulus is defined as a square becoming darker, upon which participants had to press a button as fast as possible. The color change may occur after 500 ms. Notably, the target stimuli make up only 5% of stimuli. The main variables of interest were *mean reaction time*, *standard deviation* of reaction time, *omission,* and *commission* errors.

#### Cognitive flexibility

The Trail Making test (TMT Langensteinbach version, Reitan 1958; Rodewald et al. 2012; Schuhfried 2010) administered through the VTS was used to measure cognitive flexibility. The TMT consists of two parts, with part A mostly assessing processing speed and Part B cognitive flexibility (Rodewald et al. 2012). In part A, numbers (1–25) are shown on a computer screen and the participant is requested to connect the numbers in ascending order as fast as possible. In part B, letters (A–L) are mixed in with numbers (1–13) and participants are asked to alternate between clicking a number and a letter in ascending order as fast as possible (e.g., 1—A—2—B—3—C—4—D). The *reaction time of Part A*, *reaction time of Part B,* and a *quotient* (dividing the response time of Part B by the response time of Part A) were recorded.

#### Verbal fluency

Verbal fluency was assessed with the Regensburger Word Fluency Test (RWT, Aschenbrenner et al. 2000). Within a 2-min time interval, participants had to produce as many words as possible starting with the letter “M”. Several rules had to be considered: (a) names (such as Maria, Michigan) were not permitted, (b) words of the same stem (e.g., moon, moon light, and moon cycle) were counted as one word, and (c) perseverations of words mentioned before were counted as errors. The *number of correctly produced words* was the main variable of interest.

#### Working memory

The working memory test (N-Back Verbal test; N-Back) of the VTS (Schellig and Schuri 2012; Schuhfried 2010) was used to measure verbal working memory, which requires participants to hold information in mind while continuously updating this information. Consonants are shown consecutively at the center of a computer screen for 1.5 s followed by an interstimulus interval of 1.5 s. In the 2-Back version, participants are asked to react as fast as possible by pressing a specific button when the current letter is the same as the letter presented second to last (two places before, e.g., V – T – **R** – N – **R,** then participants should press a button once the second R is shown). The duration of this task is about 9 min. The *sum of correct responses*, *omissions,* and *commissions* were recorded.

#### Planning

The Tower of London (TOL, Freiburg version) test of the VTS (Kaller et al. 2011; Schuhfried 2010; Shallice 1982) was employed as a measure of planning ability. In this test, participants are presented with a three-dimensional visualization of three wooden rods and three colorful balls (blue, yellow, and red) that need to be sorted into a specific goal state. Participants are asked to move the balls from a starting position into a goal state, which is displayed above the task imagine, using as few moves as possible. For each set, the minimum number of moves is displayed alongside the goal state. The balls can only be moved under adherence to specific rules, e.g., only one ball may be moved at a time and the rods have different lengths and may only hold a specific number of balls. Thus, to solve the task with as little moves as possible, participants need to plan ahead how to move the balls. A total of 28 sets increasing in difficulty are presented and participants have a maximum of 1 min to solve each set. This test takes about 16 min to complete. The measure of interest is a *score of planning ability*, which is computed by adding the number of the sets that were solved in the minimum amount of moves (the sets in the beginning of the test that can be solved in three moves are counted as practice trials and not included in the final score).

#### Verbal memory

To assess verbal memory, the Verbal Learning and Memory Test (VLMT, Helmstaedter et al. 2001) was used. This test measures several aspects of verbal memory: immediate recall, learning, delayed recall, and delayed recognition. A list of 15 everyday words [e.g., house, moon, river, and (immediate recall)]. The list is read out another 4 times, and after each reading, participants have to name as many words as possible (learning). Afterwards, an interference list (made of everyday words such as cherry, cloud, and glasses) is read out and participants are asked to recall all words they can remember. Next, without reading out the first list again, participants are asked to recall all words from the first list that they can remember. After a delay of 20–30 min, participants are asked again to name all words of the first list they can remember (delayed recall). Next, a series of words (including the words of the first list, the interference list and completely new words) is read out to the participants, who have to indicate with a yes or no whether the respective word belongs to the first list or not (delayed recognition). For all aspects of memory, the correctly remembered words are summed up and compared to age specific norms. The *number of correctly produced words* of the first trial constitutes the score for immediate memory, the *sum of all correct words* across the first five trials represents the score for learning, the *number of correct words* after the delay makes up the delayed recall, and the *number of correctly recognized words* of the first list (when they are mixed in with other words) represents the delayed recognition.

#### Symptom and performance validity

Credibility of patients with ADHD was assessed with two measures of symptom and performance validity. The Conners Infrequency Index (CII) was applied to detect noncredible symptom reports of patients with ADHD (Suhr et al. 2011). The CII is embedded in the long version of the CAARS (Conners et al. 1998; Suhr et al. 2011, see description of the CAARS above) and a sum *score* of 21 or higher on the CII may indicate that participants do not respond in a credible manner.

The Groningen Effort Test (GET, Fuermaier et al. 2017a, b; Fuermaier et al. 2016; Schuhfried 2010) is a performance validity test specifically designed to detect noncredible cognitive performance of adults with ADHD. It is based on the principles of an embedded figures test, wherein participants decide whether a simple target figure is contained within a more complex figure. The response time per stimulus and the number of errors per quarter were combined into an *index score*, with scores > 1 indicating noncredible performance.

### Self-evaluation of cognitive test performance

A visual aid representing a normal distribution made out of blue stick figure people was used to support the self-evaluation of cognitive performance (Supplement A). Below the bell-shaped display of people, a line is printed with numerical markings (“1” alongside with the verbal description “the worst”, “5”,”10”, “25”, “50” with the description “average”, “75”, “90”, “95”, “99” with the description “the best”), which represent the percentile ranks. Importantly, the numerical markings are unevenly spaced to match a normal distribution where points at the extremes are spread out much further than points at the center. Above the normal distribution, a reminder specifies that participants need to compare themselves with 100 people of their age. There are two versions of the visual aid, one as described above, and the other includes two example persons to demonstrate how to use the visual aid (Supplement B). Person 1 is a female printed in red and situated a bit to the right of the 75th percentile marking. A red arrow is pointing at this person and underneath the description “Person 1 Rank 81″ is added. Person 2 is a male printed in green located slightly to the right of the 10th percentile marking. A green arrow with the description “Person 2 Rank 14″ is printed underneath the person.

To ensure that participants understand how to use the visual aid, a standardized instruction was read out to the participants ( "Appendix") and both visual aids are shown to the participants. In this instruction, the rationale of a normal distribution is explained, i.e., how most people perform averagely and are clustered around the center of the distribution and how only a few people perform very well or very badly and form the extremes. It is mentioned how this relates to the numerical markings of the percentile ranks printed underneath the visual aid. Then, the example persons are introduced to demonstrate the concept of percentile ranks. Participants are given an opportunity to ask questions and given two practice questions. To check whether the participants understood the concept of percentile ranks, they are asked how many people perform worse and how many people better than them given the rank they indicated. If a wrong answer is given, the rationale of percentile ranks is explained again.

## Procedure

Before the start of the assessment, the participants read through an information sheet and gave their written informed consent. Each person was tested individually and no financial compensation was offered. The anamnesis was performed in the beginning of the assessment, followed by the verbal intelligence estimate. Next, the instruction of the self-evaluation of cognitive performance was read out to the participants and the visual aid was introduced. Once the participants answered the practice questions appropriately, the neuropsychological test battery was administered. To prevent order effects, the test sequence was alternated. Five different test sequences of the neuropsychological battery were created for this purpose and for each participant, one of the sequences was randomly selected. The sequences were designed, so that in the 20-min delay of the verbal memory test, only nonverbal tests were administered to prevent interference with the verbal memory test. For all neuropsychological tests, the participants completed the respective test and then were asked to reflect on their test performance and evaluate how well they performed relative to their same aged peers (postdiction self-evaluation of cognitive performance). The self-evaluation questions for each particular test were standardized ("Appendix") to tailor them as much as possible to the task parameters. In addition, questions were phrased to ensure that they relate to salient characteristics of the tests, so that participants have a clear idea what characteristics to evaluate themselves on. For example, the question for the NBV (outcome variable: sum of correct letters) was: “Compared to others, how well could you remember the letters?”. After the participants had completed all neuropsychological tests, they were asked to fill in the questionnaires. The questionnaires of the relatives of the participants could either be filled out before the testing day and brought along to the assessment, or filled in afterwards and sent in. At the end of the assessment, the participants were debriefed. The total duration of the assessment was around 3 h per participant and included the completion of the test battery and the questionnaires mentioned above as well as other neuropsychological measures part of the larger research project.

## Ethics statement

Ethical approval (S-383/2010) was obtained from the ethics committee of the medical faculty of the University of Heidelberg, Germany. The study was conducted in adherence with the ethical standards of the Helsinki Declaration (7th revision, 2013).

## Statistical analysis

First, means and standard deviations of all measures of neuropsychological test performance and self-reports of cognitive performance (FLei) were calculated separately for patients with ADHD and controls. As the normality and homoscedasticity assumptions were violated in several instances, nonparametric analyses were chosen. To establish the presence or absence of neuropsychological deficits in the patient group compared to controls, Mann–Whitney *U* tests were computed and effect sizes were calculated. For all analyses, the effect size Cohen’s r was chosen as it does not rely on the normality or homoscedasticity assumptions. According to Cohen (1988), the size of the effect was classified as small (0.1 ≤ r < 0.3), medium (0.3 ≤ r < 0.5), or strong (r ≥ 0.5). No correction of the alpha error inflation was applied as the main goal of this study is an exploration of metacognition in adult ADHD, and due to this broad focus, it seemed appropriate to investigate a number of cognitive domains and functions using *p* < 0.05. A strict alpha-level correction such as the Bonferroni method might have resulted in missing effects (type-2 errors), yet not applying any correction represents an important limitation (risk of type-1 errors) and will be elaborated on in the discussion section. Consequently, effect sizes (independently of significance levels) will be the basis of interpretation for the results.

Second, discrepancy scores (DS) of neuropsychological test performance were calculated. For all neuropsychological tests applied, the actual percentile rank was subtracted from the postdiction percentile rank, i.e., the percentile rank of the actual test performance (as indicated by age-based norms) was subtracted from the self-evaluated percentile rank (based on the visual aid). A positive DS would indicate that the participant overestimated his or her performance, while a negative DS would indicate that the participant underestimated his or her performance. To compare metacognition across neuropsychological domains, DS were grouped in domains based on which neuropsychological functions the DS are based on. Thus, the attention domain encompasses the DS of *vigilance RT, vigilance omissions,* and *selective attention*. The domain of executive functions includes the DS of *processing speed, cognitive flexibility, verbal fluency, working memory,* and *planning*. For the memory domain, the DS of *immediate recall, learning, delayed recall,* and *recognition* were included. Next, mean DS per domain were calculated by averaging each individual DS of the functions of each domain. Furthermore, a total DS was computed for each individual by averaging the DS across the 3 domains. To compare patients with ADHD and controls, the means and standard deviations for each DS were calculated separately. Next, Mann–Whitney *U* tests as well as effect sizes were computed to explore whether patients differed from controls in their self-awareness (DS) of cognitive performance. To assess whether there are differences between cognitive domains, Mann–Whitney *U* tests and effect sizes were calculated for the DS of the 3 individual domains as well as the total DS of cognitive performance.

For the self-report and informant-report of cognitive performance, DS were calculated by subtracting the self-rating from the informant-rating for each of the Flei subscales and for the total score. Mann–Whitney *U* tests and effect sizes were computed to assess differences between patients with ADHD and controls on the resulting DS. To investigate the role of depressive symptomatology in self-awareness, the domain and total DS of neuropsychological test performance were correlated (Spearman rank correlation coefficients) with the BDI.

## Results

Descriptives of self- and informant-reports of cognitive functioning are reported in Table 1 and of neuropsychological test performance in Table 2 (Appendix). Compared to controls, patients with ADHD were significantly slower in their reaction time on the vigilance (*p* = 0.002; *r* = 0.315) and selective attention (*p* = 0.002; *r* = 0.316) tasks and made more omission errors in the vigilance task (*p* < 0.001; *r* = 0.436). These effects were medium in size. No significant differences were found between patients and controls on any of the executive functioning tests (*r* ranging from 0.053 to 0.181). Patients scored significantly lower than controls on all memory functions, with small effects for immediate recall (*p* = 0.021; *r* = 0.239) and recognition (*p* = 0.044; *r* = 0.208) and medium effects for learning (*p* = 0.002; *r* = 0.325) and delayed recall (*p* = 0.002; *r* = 0.312).

Analyses of the DS are displayed in Table 3 (Appendix). Comparisons of the DS of self- and informant-report revealed no significant differences between patients with ADHD and controls (*r*: ranging from 0.003 to 0.162). The DS of cognitive performance showed that patients with ADHD significantly overestimate their attentional functions (Attention DS: *p* = 0.014; *r* = 0.255) as compared to controls. This effect is small. Considered individually, no statistically significant difference was found for the omission errors on the vigilance task. For vigilance RT and selective attention, patients overestimated their cognitive performance with a small effect (Vigilance RT: *p* = 0.011; *r* = 0.263; Selective attention: *p* = . 031; *r* = 0.224) as compared to controls. For the executive functions (*r*. ranging from 0.016 to 0.121) and memory domain DS (*r*. ranging from 0.023 to 0.096) including all respective functions, no significant differences were found between patients and controls. The number of participants overestimating, underestimating, and correctly estimating their performance showed that on 9 out of 12 DS of neuropsychological functions, patients with ADHD overestimated their performance and controls overestimated their performance on 7 out of 12 measures.

To explore whether the current findings may have been affected by depressive symptomatology in patients with ADHD versus controls, Spearman’s rho correlations were computed between BDI scores and both domain DS and total DS of neuropsychological test performance. In patients with ADHD, a medium correlation (*p* = 0.027; *rho* = 0.322) between the total DS of neuropsychological test performance and BDI was found. The other correlations did not reach statistical significance (attention DS, executive functions DS and memory DS with BDI; *p values* ranging from 0.084 to 0.494; *rho coefficients* ranging from 0.102 to 0.255). In controls, none of the DS of neuropsychological test performance correlated with the BDI (*p values* ranging from 0.326 to 0.925; *rho coefficients* ranging from 0.014 to 0.150).

## Discussion

The aim of the current study was to explore metacognition in adults with ADHD. The first step was to establish the extend of cognitive impairment in attention, executive functions, and memory in adult patients with ADHD as compared to controls. The results indicate that deficits are surprisingly clear cut by domain: relative to controls, patients with ADHD show impairments in attentional functions (Table 2; medium effects) and in memory functions (small-to-medium effects), whereas in executive functions, no significant impairment was found (negligible to small effects). These deficits in attention and memory are in line with previous research (Schoechlin and Engel 2005). The lack of significant deficits in executive functions is slightly surprising and one may speculate whether matching patients with controls on IQ may have affected the results, as IQ may moderate executive functioning impairment in adults with ADHD (Antshel et al 2010). Yet, whether or not deficits in executive functions are found may also be highly dependent on exactly which executive functions are tested and with which neuropsychological tests (Thome et al. 2012). The evidence base for executive function deficits in adults with ADHD is rather heterogeneous, with merely a portion of patients displaying deficits in executive functions (e.g., 31%; Biederman et al. 2006). Whereas only a minority of adults with ADHD may show impairments in executive functioning tests, on self-reports of executive functions, most adults with ADHD indicate impairments (Barkley et al. 2011). This is mirrored in the present study: whereas no impairment could be found on tests of executive functions, patients rated their executive functions as impaired.

The next step was to compare patients’ and controls’ self-evaluation of cognitive performance with their actual performance on neuropsychological tests. Adults with ADHD were expected to differ from controls in their self-awareness of cognitive performance. Significant differences were found in self-awareness of attention but not in self-awareness of executive functions or memory (Table 3). Patients with ADHD were impaired on attentional functions relative to controls, but rated their performance on these tasks higher than controls (small effects). It is important to note that for the reaction time on the vigilance task, patients with ADHD were actually more accurate in their self-evaluation than controls (small effect). Nevertheless, if all attentional functions are considered together, patients with ADHD show a deficit in metacognition in the attentional domain (small effect). For executive functions, patients with ADHD showed no impairment in test performance and also rated their performance similarly to controls. This extends the findings by (Knouse et al. 2006) who found deficient self-awareness of driving ability in adults with ADHD. The current results suggest that this deficiency in self-awareness of driving ability may be due to impaired self-awareness of attentional but not executive functions. For memory, although adults with ADHD showed impairments on all memory tasks, they seemed aware of these deficits. This aligns with findings by (Knouse et al. 2006), who investigated meta-memory and found no difference between adults with ADHD and controls in their judgement of learning, implying that adults with ADHD may be able to judge their memory performance as realistically as controls. Our second prediction stipulated a difference in self-awareness between cognitive domains for patients with ADHD relative to controls, and indeed, patients showed self-awareness deficits in one domain but not in the other two domains. Patients with ADHD and controls did not differ in their self-awareness of executive functions and memory performance, implying that these aspects of metacognition seem intact in patients with ADHD. In contrast, the deficit in self-awareness of attentional functions is rather striking as ADHD is known for and characterized by attentional deficits (Hervey et al. 2004), whereas impaired memory functioning is not common knowledge.

Several explanations could be brought forward why patients with ADHD showed a deficit in self-awareness of attentional functions but not in self-awareness of memory. First, addressing the face validity of tests, one could argue that remembering a list of words is closer to memory tasks of daily life (e.g., remembering which groceries to get from the supermarket) than is the response to abstract stimuli on a computer screen to attention tasks of daily life. Thus, increasing the face validity of the test may facilitate participants’ self-assessment, so that they may be able to draw from real live examples to evaluate their cognitive performance. Moreover, the availability of feedback may affect participants’ self-awareness of cognitive performance. For example, in the memory task of the present study, the same word list is repeatedly read out, enabling participants to derive feedback regarding their performance. Task parameters such as indicators of task success and repeated exposure were shown to influence self-awareness accuracy in nonclinical samples (Rothlind et al. 2017). Accordingly, the issues of face validity and feedback may also apply to control participants (Rothlind et al. 2017). Another aspect affecting self-awareness in patients with ADHD may be task complexity. In more complex tasks (e.g., Tower of London, N-Back), patients often reported difficulties after test administration. Thus, patients seem to be aware of the cognitive demands in more complex tasks in that they notice they are experiencing difficulties (e.g., cognitive fatigue, difficulty concentrating) keeping up with those demands. This may guide them to assess their cognitive abilities more realistically. In simple tasks, on the other hand, it may not be noticeable for the patient that they performed poorly, and thus, they may not be aware of any deficits. It is worth noting that on most measures, (12 out of 16) patients with ADHD showed a trend to overestimate their abilities as compared to controls. It would be interesting to replicate this study with a larger sample and more sensitive measures to explore this tendency of patients with ADHD to overestimate themselves.

Finally, it was explored whether any difference in metacognition between patients and controls measured by the postdiction discrepancy method could also be found by subjective accounts (self-report and informant-report) of cognitive functioning. DS of subjective experience of cognitive deficits based on self-report and informant-report revealed no difference between patients with ADHD and controls (Table 3). This implies that patients’ perceptions of their cognitive capabilities and their relatives perceptions largely correspond to the perception of controls and their relatives. As previous research indicated substantial discordance between subjective (self-report) and objective (neuropsychological testing) approaches (Barkley et al. 2011; Fuermaier et al. 2015), we did not expect the information yielded from the two sources to converge. Indeed, the reduced self-awareness of patients with ADHD of their attentional deficits that was found with the postdiction discrepancy method was not found on subjective accounts (self-report and informant-report) of cognitive functioning. In extension to the finding that executive function tests and self-reports do not seem to converge (Barkley and Murphy 2010; Fuermaier et al. 2015), the current results indicate that informant-report may not correspond with test performance either, as relatives of patients with ADHD rated the patients’ cognitive functioning in all domains as impaired (Table 1). Relatives may struggle to evaluate the patient’s cognitive processes accurately and they may rely on the patients’ reports and outward expressions of their experience, which may not be accurate, particularly given the current results indicating self-awareness deficits in attention. It would be interesting for future research to investigate this in more depth, for example by exploring whether there is a difference in the accuracy of informants’ evaluations between more readily observable functional outcomes and cognitive test performance.

## Clinical implications

Interestingly, a number of studies discussed the utility of metacognitive therapy in students (Teixeira Pisacco et al. 2018; Thompson and Thompson 1998) and adults with ADHD (Solanto et al. 2010; Teixeira Pisacco et al. 2018; Thompson and Thompson 1998; Wasserstein and Lynn 2001), even though it had not been established yet whether deficits in metacognition really exist in adults with ADHD. One may raise the question to what extend these therapies indeed target and improve “metacognition” (Wells and Fisher 2011). Upon closer inspection, these “metacognitive” therapies include cognitive training such as breaking down tasks, rather than targeting metacognition (Wells and Fisher 2011). To address metacognitive awareness, clinicians could for example actively encourage patients to reflect on their cognitive strengths and weaknesses. To aid this process, the clinician could give the patient specific feedback on their cognitive performance, so that the patient may adjust his or her evaluation, as awareness of one’s deficits may be the first step in an effective treatment trajectory (Williamson et al. 2010). To optimally target treatment to the needs of adult patients with ADHD, it is essential to first establish an evidence base for metacognitive functioning in adults with ADHD. The current study was one of the first steps in that direction by demonstrating that there are indeed deficits in self-awareness in adult ADHD and that these deficits may be domain specific. Future research could try to identify mechanisms that can help to compensate any deficits, possibly exploring the effects of daily relevance of tasks, feedback, and task complexity. For example, the results of this study suggest that if feedback is available during task execution, patients may be able to use this to adjust their self-evaluation. In a more complex task, patients may notice “internal feedback” of struggling with the task, getting tired and so forth and may adjust their evaluations accordingly. This could indicate that feedback helps patients to engage in the self-monitoring aspect of metacognition, which is worth exploring in future research. For the clinical evaluation of patients with ADHD, it may be important to consider that patients may lack awareness of their deficits in attentional tasks and may overestimate their abilities. Therefore, if, for example, decisions regarding driving need to be made, clinicians may want to contemplate the combination of deficits in self-awareness of attention functions and driving ability as well as the increased presence of unsafe driving behaviors in adults with ADHD (Fuermaier et al. 2017a, b; Knouse et al. 2005). Once more information is available on how metacognitive functioning is affected in adult ADHD, metacognitive interventions could be developed that identify individual cognitive strengths and weaknesses, give feedback and employ strategies that capitalize on the individuals’ cognitive strengths. Furthermore, the current results showed a lack of correspondence between subjective and objective measures of metacognition. Given the widespread reliance on self-report, this has direct implications for the clinical assessment, particularly for self-reported attention deficits. It may be wise for clinicians to keep in mind that any given patient with ADHD may be unaware of their deficits in attention and/ or overestimate their abilities and thus may have larger impairments than what is indicated based on patients’ own evaluation.

## Limitations and directions for future research

Several limitations and suggestions for future research should be noted. Although this study aimed to explore metacognition in adults with ADHD, the scope of the methodology covers the self-awareness (discrepancy between estimated and actual performance) aspect of metacognition, but does not capture the self-monitoring and self-regulation elements. To gain a comprehensive picture of metacognitive functioning in adult ADHD, it may be worthwhile for future research to explore other aspects of metacognition. As deficits in motivation regulation have been implicated in adult ADHD (Volkow et al. 2011), one may speculate that self-regulation could be problematic for patients with ADHD. In addition, in schizophrenia, metacognition and motivation seem to affect each other (Luther et al. 2016; Tas et al. 2012), and as motivation has been implicated in ADHD, it would be relevant for future research to explore how metacognition relates to motivation in adults with ADHD. Another limitation is the abstract nature of cognitive tests, rendering them far removed from peoples’ real-life experience. Even for healthy controls, it may be hard to realistically assess their cognitive abilities on these tests, as, for example, the DS of both patients with ADHD as well as controls in executive function were elevated, indicating that both groups were struggling to assess their executive functions accurately. Maybe, patients with ADHD have subtle deficits in metacognition that are present in daily life, but may not have been captured by tests designed to assess impairment in neuropsychological performance. Future research could employ tasks that resemble real-life more closely to relate self-awareness based on cognitive testing to functional outcomes. Another point of concern is the high prevalence of comorbidities in the patient group. In the current study, 53% of patients were diagnosed with a mood disorder and 31% with an anxiety disorder (the percentages are not cumulative as some patients were diagnosed with both). Research on prevalence of comorbidity in patients with ADHD found roughly comparable figures, albeit a slightly lower prevalence rate of depression (38–42%) and higher rate of anxiety disorders (45–47%, Chen et al. 2018; Kessler et al. 2006). Regarding comorbidities in general, 71.9% of adults with an ADHD diagnosis may have an additional DSM-IV-TR axis I disorder (most commonly mood disorders and anxiety disorders) and 50.9% a comorbid DSM-IV-TR axis II disorder (Cumyn et al. 2009). Accordingly, the incidence rate of comorbidities in the current sample is roughly in line with research investigating the prevalence of comorbidities in adult ADHD and, therefore, should not limit the generalizability of the current results to the ADHD patient population. It is worth noting that there may be a complex interplay between metacognition and depressive symptomatology, and many patients in the current sample were diagnosed with comorbid depression. To shed light on whether the current findings may have been confounded by depressive symptoms, correlation analyses were conducted. No significant association between the deficit in self-awareness of attentional functions and scores on the BDI was found, indicating that the main finding of the current study does not seem be affected by depressive symptomatology. Some of the patients with ADHD in the present study were treated with stimulants and a large number of patients with antidepressants, which may have had affected their ability to reflect on their own performance. Future research could explore metacognitive awareness before and after antidepressant and stimulant treatment to shed light on to what extent these medications affect patients’ self-awareness. Regarding the statistical analysis, an important limitation is the lack of a statistical control for alpha error inflation in multiple testing. Due to the exploratory nature of this paper, the authors decided not to apply an alpha correction such as the Bonferroni method to avoid type II errors (false negatives). This, however, comes at the cost of risking Type I errors (false positive). It is important to consider that results may have been affected by the size of the sample (*N* = 47 for each group), which may limit generalizability. While this study serves as a first indication of the presence of metacognitive difficulties in adults with ADHD, a replication of this study with a larger sample and more sensitive measures would be advisable. The selection of neuropsychological measures also influenced the findings, and therefore, it would be worth replicating the study with a different selection of neuropsychological tests and questionnaires assessing the same constructs. Finally, the quasi-experimental approach relying on matched groups comes with concerns regarding internal validity and the conclusions drawn should be interpreted in this light.

## General conclusion

To conclude, adults with ADHD show deficits in self-awareness of attentional functions, but seem to have intact metacognitive awareness in memory and executive functions. The current results indicate that face validity, feedback, and task complexity may be important factors affecting patients’ ability to reflect on their own cognitive processes. This warrants further research to elucidate which factors may impede and which factors may help patients with ADHD utilize and improve their metacognitive skills. This information may be applied in a clinical context to tailor treatment interventions. Subjective (self- and informant-report) and objective (DS of self-evaluated and actual test performance) accounts of metacognition do not seem to correspond, at least not in areas in which the patients with ADHD show deficits in self-awareness. Furthermore, patients seem to be unaware of their deficits in attention and have a tendency to overestimate their cognitive abilities, which could be important to consider in a clinical context. Further research is needed to investigate if patients with ADHD also have deficits in the self-monitoring and self-regulation aspect of metacognition and also how any deficits in metacognition relate to functional outcomes and the struggles patients face in their everyday life.

## Supplementary Information

Supplementary file 1 ( DOCX 709 kb )

Supplementary file 2 ( DOCX) 776 kb )

## Data Availability

Data can be accessed from the authors upon reasonable request.

## Appendix 1

See Tables 1, 2, and 3.

**Table 1:** Characteristics of patients with ADHD and controls (mean ± standard deviation)

	Patients with ADHD (*N* = 47)	Controls (*N* = 47)
Age (years)	35.79 ± 11.07	34.72 ± 13.66
Intellectual functions (IQ)^a^	104.36 ± 10.89	105.85 ± 10.90
Educational level^b^	1.83 ± 1.36	1.45 ± 1.08
Gender (female/male)	19/28	25/22
ASRS^c^	28.40 ± 8.17*	10.22 ± 6.25
WURS-K^d^	36.18 ± 12.89*	13.50 ± 9.62
BDI^e^	20.21 ± 10.71*	6.91 ± 6.95
Flei Attention^f^	26.24 ± 8.19*	9.27 ± 6.23
Flei Executive functions^f^	21.51 ± 8.13*	8.98 ± 5.83
Flei Memory^f^	24.80 ± 7.32*	11.82 ± 6.25
Flei Total^f^	72.55 ± 21.59*	30.07 ± 17.21
I-Flei Attention^g^	20.54 ± 9.39*	6.47 ± 6.21
I-Flei Executive functions^g^	19.50 ± 9.00*	5.67 ± 4.66
I-Flei Memory^g^	17.74 ± 9.40*	7.31 ± 5.38
I-Flei Total^g^	57.78 ± 25.71*	19.44 ± 15.0

^*^ Pairwise comparison significant at p < .001

^a^ Multiple Choice Vocabulary Test (MWT-B)

^b^ Lower scores indicate higher educational level

^c^ ASRS: ADHD self-rating scale

^d^ WURS-K: Wender Utah rating scale, short form

^e^ Beck’s Depression Inventory

^f^ Self-report version of the complaints of cognitive disturbances questionnaire (Flei)

^g^ Informant-report version of the complaints of cognitive disturbances questionnaire (Flei)

**Table 2:** Cognitive performance based on neuropsychological testing (mean ± standard deviation)

Measure	Patients with ADHD (*N* = 47^ m^)	Controls (*N* = 47^ m^)	*MW-U*	*p*	Cohen’s *r*
Vigilance RT^a^	443.64 ± 85.21	394.26 ± 67.48	701.00	**.002**	**.315**
Vigilance omissions^b^	1.83 ± 2.72	.26 ± .71	629.00	**.000**	**.436**
Selective attention^c^	401.89 ± 94.65	346.55 ± 66.94	699.50	**.002**	**.316**
Processing speed^d^	18.21 ± 3.99	17.57 ± 6.20	872.50	.079	.181
Cognitive flexibility^e^	32.44 ± 12.83	33.62 ± 33.20	883.00	.094	.173
Verbal fluency^f^	15.89 ± 4.39	17.43 ± 6.22	932.00	.191	.135
Working memory^g^	11.39 ± 2.70	11.98 ± 3.49	855.50	.080	.181
Planning^h^	15.02 ± 3.04	15.17 ± 3.77	1015.00	.610	.053
Immediate recall^i^	6.91 ± 1.84	8.06 ± 2.67	802.50	**.021**	**.239**
Learning^j^	52.53 ± 10.70	58.45 ± 8.31	688.00	**.002**	**.325**
Delayed recall^k^	11.09 ± 2.81	12.72 ± 2.48	708.50	**.002**	**.312**
Recognition^l^	14.06 ± 1.26	14.55 ± .75	870.00	**.044**	**.208**

**bold = **significant at* p* < 0.05

^a^Mean reaction time in ms in the vigilance task (WAFV); ^b^Total number of omission errors in the vigilance task (WAFV), ^c^Mean reaction time in ms in the selective attention task (WAFS); ^d^Total response time in seconds in the Trail Making Test Part A (TMT-A); ^e^Total response time in seconds in the Trail Making Test Part B (TMT-B); ^f^Sum of correctly produced words in the verbal fluency test (RWT); ^g^Sum of correctly identified letters on the N-Back task (N-Back); ^h^Composite score based on optimally solved items in the Tower of London test (TOL); ^i^Number of correctly recalled words on the first trial on the verbal learning test (VLMT); ^j^Sum of correctly recalled words across the first five trials on the VLMT; ^k^Number of correctly recalled words on the seventh trial on the VLMT; ^l^Number of correctly identified words on the recognition part of the VLMT; ^m^For the planning and working memory tasks, data of one participant was missing (*N* = 46)

**Table 3:** Discrepancy scores (DS) of self-report and cognitive performance

Discrepancy scores		Patients with ADHD	Controls	Statistics		
				*MW-U*	*p*	Cohen’s *r*
Self-report *(DS* = *raw score self-rating – raw score informant-rating*^*q*^*)*						
Attention	DS	6.27 ± 11.93	2.80 ± 7.31	712.00	.137	.162
Executive functions	DS	2.77 ± 11.60	3.31 ± 6.70	874.50	.979	.003
Memory	DS	7.41 ± 9.63	4.51 ± 7.61	721.00	.160	.153
Self-report total	DS	16.44 ± 31.82	10.62 ± 19.79	763.50	.306	.112
Neuropsychological test performance *(DS* = *PR postdiction – PR actual performance)*						
Vigilance RT^a^	N	22/22/2	16 / 31 / 0			
	DS	2.91 ± 28.97	−10.19 ± 29.51	751.50	**.011**	**.263**
Vigilance omissions^b^	N	31/16/0	21/25/0			
	DS	12.81 ± 37.93	1.07 ± 28.22	865.00	.097	.172
Selective attention^c^	N	34/12/0	29/17/1			
	DS	22.11 ± 26.37	10.32 ± 26.62	799.50	.**031**	**.224**
Attention DS	DS^d^	11.74 ± 24.57	.45 ± 22.44	778.00	**.014**	**.255**
Processing speed^e^	N	37/10/0	27/19/1			
	DS	21.28 ± 26.53	13.96 ± 31.03	950.00	.243	.121
Cognitive flexibility^f^	N	29/18/0	26/21/0			
	DS	14.45 ± 30.66	7.53 ± 35.39	967.00	.298	.107
Verbal fluency^g^	N	34/12/1	29/18/0			
	DS	9.98 ± 16.84	5.13 ± 22.20	981.50	.352	.096
Working memory^h^	N	24/21/0−1.96 ± 21.40	27/20/0 2.34 ± 31.21			
	D S	−1.96 ± 21.40	2.34 ± 31.21	923.00	.293	.110
Planning^i^	N	23/22/0	27/20/0			
	DS	7.84 ± 29.30	8.70 ± 31.11	1037.50	.876	.016
Executive functions	DS^j^	10.37 ± 16.45	7.53 ± 18.96	1010.50	.477	.073
Immediate recall^k^	N	8/36/3	7/39/1			
	DS	−20.62 ± 22.75	−21.31 ± 24.78	1074.50	.820	.023
Learning^l^	N	18/28/1	10/ 32/ 5			
	DS	−7.85 ± 28.56	−11.23 ± 20.74	1019.50	.520	.066
Delayed recall^m^	N	27/20/0	20/ 25/ 2			
	DS	−1.02 ± 25.48	−1.09 ± 21.07	1057.00	.719	.037
Recognition^n^R	N	22/21/4	30/14/3			
	DS	2.17 ± 27.08	6.19 ± 25.18	981.50	.352	.096
Memory	DS^o^	−6.83 ± 19.83	−6.86 ± 13.21	1089.00	.907	.012
Test performance total	DS^p^	5.09 ± 14.95	.37 ± 13.69	889.50	.104	.168

DS (Self-report) = raw score self-rating – raw score informant-rating; DS (Neuropsychological test performance) = percentile rank postdiction – percentile rank actual performance; N = Number of participants overestimating/ underestimating/ perfect estimating; **Bold = **significant at p < 0.05

^a^Age based percentile rank (PR) mean reaction time in ms in the vigilance task (WAFV); ^b^PR of the total number of omission errors in the vigilance task (WAFV), ^c^PR mean reaction time in ms in the selective attention task (WAFS); ^d^Computed by averaging across all attention DS; ^e^PR total response time in seconds in the Trail Making Test Part A (TMT-A); ^f^PR total response time in seconds in the Trail Making Test Part B (TMT-B); ^g^PR sum of correctly produced words in the verbal fluency test (RWT); ^h^PR sum of correctly identified letters on the N-Back task (N-Back); ^i^PR composite score based on optimally solved items in the Tower of London test (TOL); ^j^Computed by averaging across all executive functioning DS; ^k^PR number of correctly recalled words on the first trial on the verbal learning test (VLMT); ^l^PR sum of correctly recalled words across the first five trials on the VLMT; ^m^PR number of correctly recalled words on the seventh trial on the VLMT; ^n^PR number of correctly identified words on the recognition part of the VLMT ^o^Computed by averaging across all memory DS; ^p^Computed by averaging across the domain DS; ^q^For the self-report N = 45, for informant report N = 39, thus 39 discrepancy scores were calculated

## Self-evaluation

Today I will ask you to complete several tests on the computer, which assess your attention and concentration. The specific tests will be explained in more detail later on.

In addition, **after** each test, I will ask you to evaluate your own performance as accurately as possible. Please compare yourself **with people of your age in the general population**. Estimate your performance for every test anew, independent of the previous tests. Please show me your estimate for every test by pointing on this visual aid.

Here *(the assessor presents the figure shown in Appendix A),* you can see the distribution of 100 persons of your age. On the scale below *(the assessor points to the scale below the figure in Appendix A)*, you can see numbers from 1 to 100, which correspond to the ranks of these 100 people.

In the tests, most people have an average performance and thus are in the middle of the distribution *(the assessor points to the middle of the distribution in Appendix A).* A few persons perform very well *(the assessor points to the right tail of the distribution in Appendix A)* and a few others very badly *(the assessor points to the left tail of the distribution in Appendix A)*. Because so many people perform averagely and only a few perform extremely well or badly, the distance between the numbers differs in size.

For example, on this graphic *(the assessor presents the figure shown in Appendix B)*, you can **see Person 1 in red** and **Person 2 in green**. Person 1 thought that she did quite well on the test and estimated herself to be on rank 81. That means that 19 people were better than Person 1 and 80 were worse. Person 2 thought that he had some difficulties with this test and because of that rated himself to be on rank 14. That implies that 86 people were better than Person 2 and 13 were worse.

Do you have any questions about this?

To practice, please answer using the visual aid. Please point out on the scale how you estimate your performance compared to others of your age.

- Compared to others, how well can you swim?

How many people are better than you and how many worse? (100 in total).

- Compared to others, how fast are you in a 100 m sprint?

How many people are better than you and how many worse?

## WAFV

1. Compared to others, ***how fast*** were you in this test? *(RT)*
2. Compared to others, ***how accurately*** did you react to the color changes? *(Omission).*

## WAFS

Compared to others, ***how fast*** were you in this test? *(RT).*

## TMT

1. Compared to others, ***how fast*** were you in this test? *(RT Trails A)*
2. Compared to others, ***how fast*** were you in this test? *(RT Trails B)*

## TOL

Compared to others, ***how well*** could you solve the exercises? *(Planning ability).*

## RWT

Compared to others, ***how well*** could you generate words? *(N correct).*

## NBV

Compared to others, ***how well*** could you remember the letters? *(N correct).*

## VLMT

1. Compared to others, ***how well*** could you memorize the words? *(First trial)*
2. Compared to others, ***how well*** could you learn the words after you heard them repeatedly? (*Learning sum,* after trial 5)
3. Compared to others, ***how well*** could you remember the words? *(Delayed recall)*
4. Compared to others, ***how well*** could you recognize the words, when they were mixed in with others? *(Recognition)*

## GET

Compared to others, ***how accurately*** could you identify the figures? *(total errors).*
