# Supplementary material for: Metacognition in adult ADHD: subjective and objective perspectives on self-awareness of cognitive functioning
Source: J Neural Transm (Vienna). 2021 Jan 19;128(7):939–55. doi: 10.1007/s00702-020-02293-w (PMC8295131; doi:10.1007/s00702-020-02293-w)
Supplement: Supplementary file 1 — ( DOCX 709 kb ) [file 702_2020_2293_MOESM1_ESM.docx]

**Supplement A: Visual aid for self-evaluation**

**Compared to 100 people of your age, you are better than…**


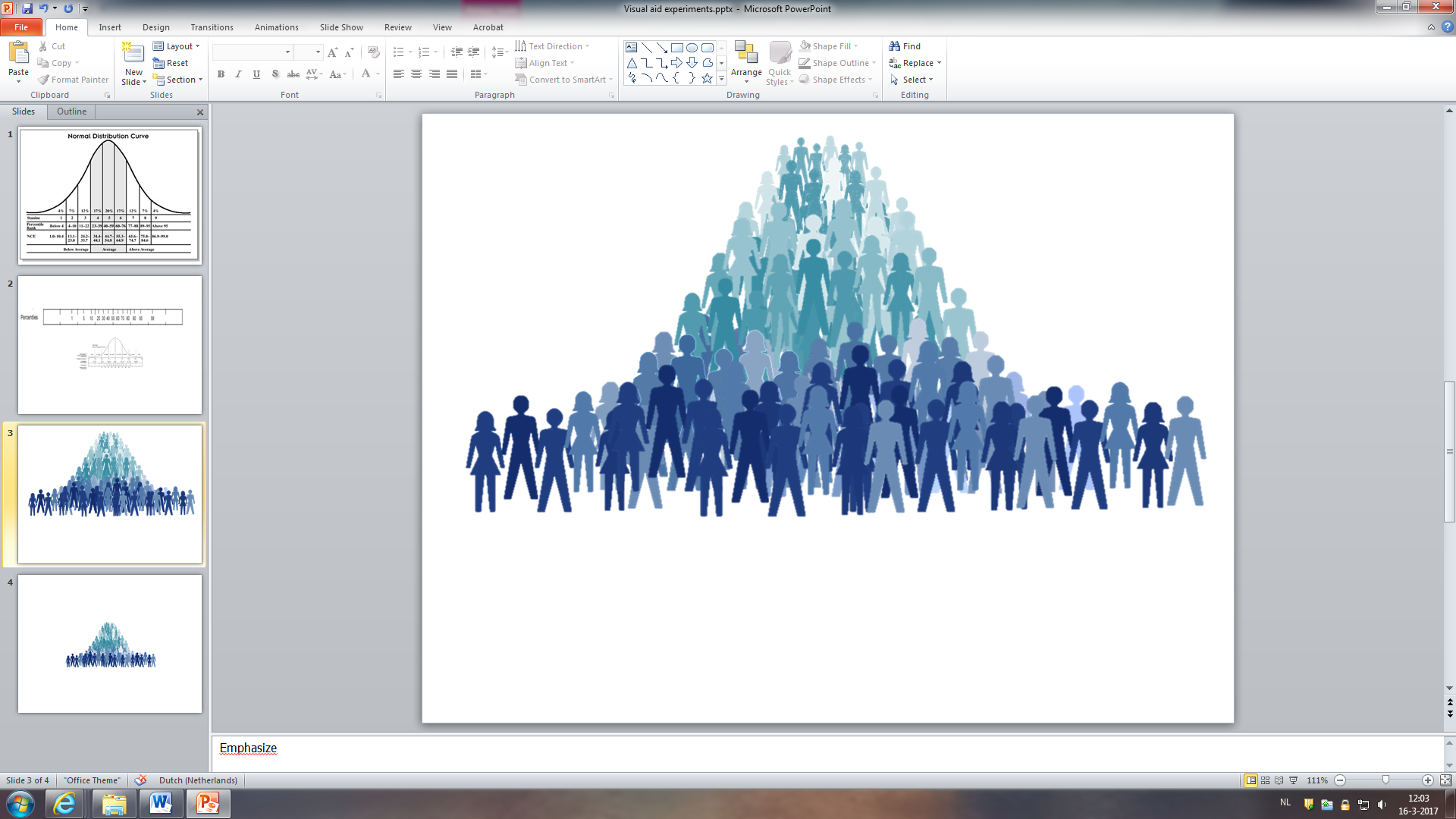

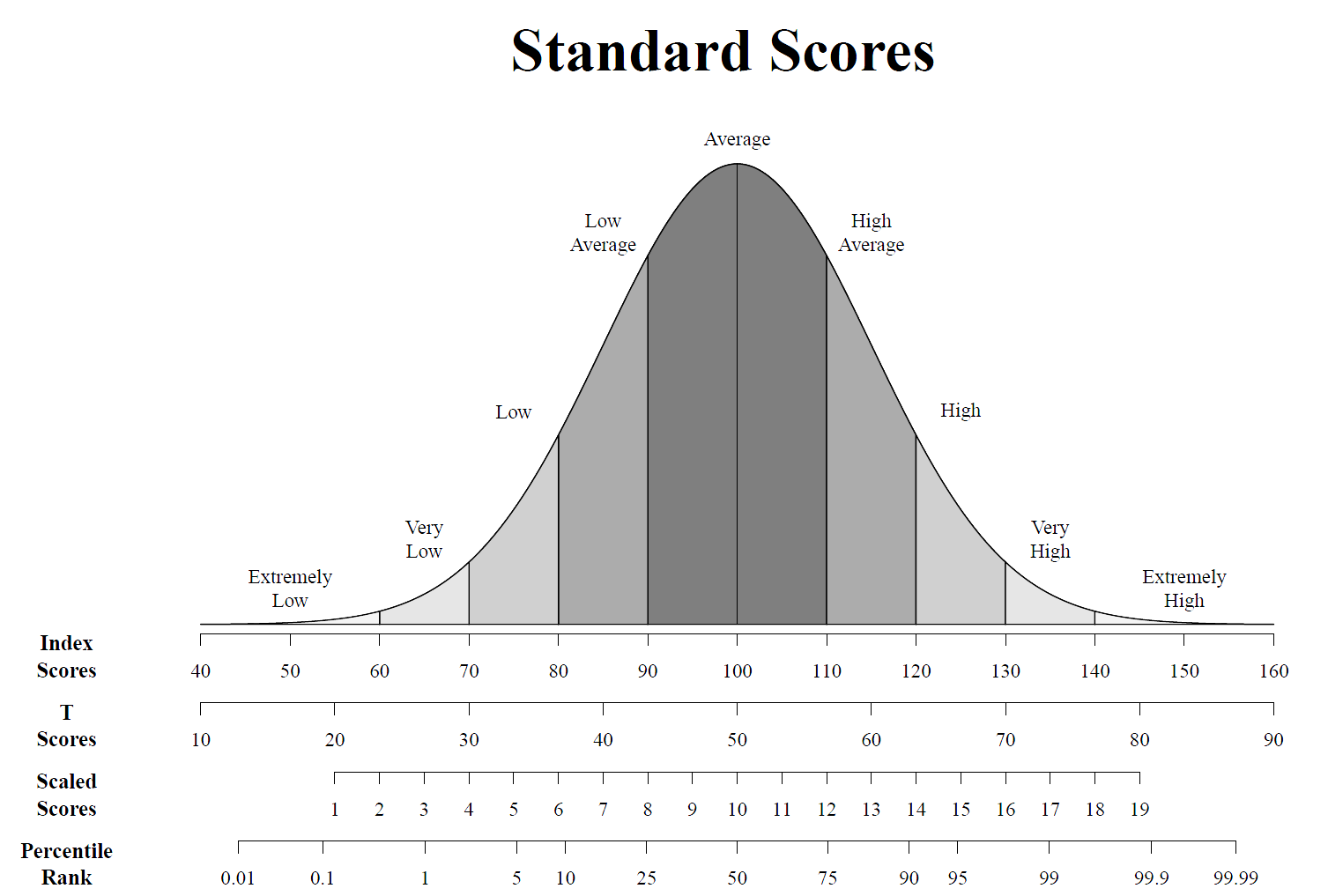


**The Average The Worst Best**
