# Supplementary material for: Metacognition in adult ADHD: subjective and objective perspectives on self-awareness of cognitive functioning
Source: J Neural Transm (Vienna). 2021 Jan 19;128(7):939–55. doi: 10.1007/s00702-020-02293-w (PMC8295131; doi:10.1007/s00702-020-02293-w)
Supplement: Supplementary file 2 — ( DOCX) 776 kb ) [file 702_2020_2293_MOESM2_ESM.docx]

**Supplement B: Visual aid for self-evaluation with examples**

**Compared to 100 people of your age, you are better than…**


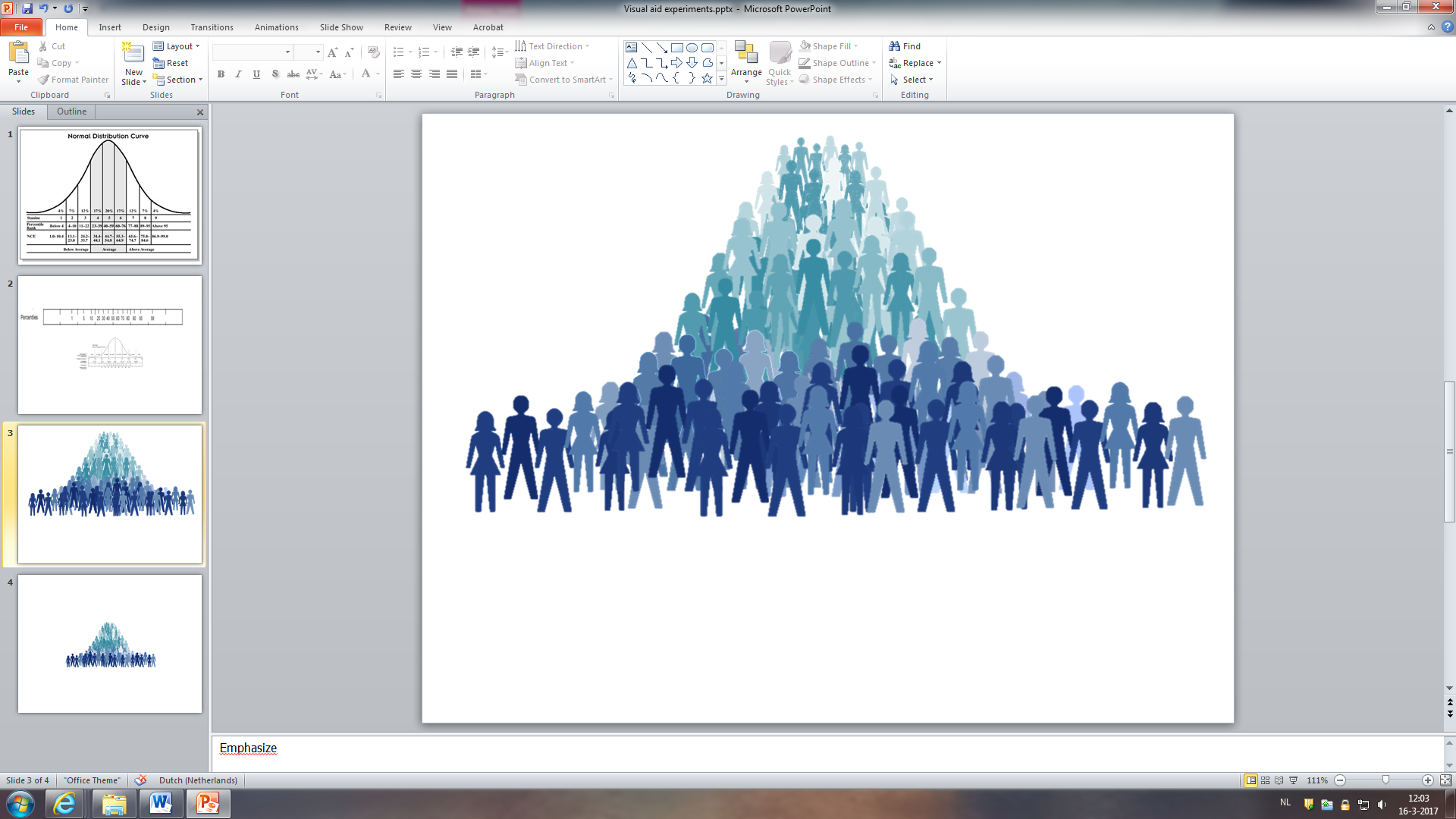


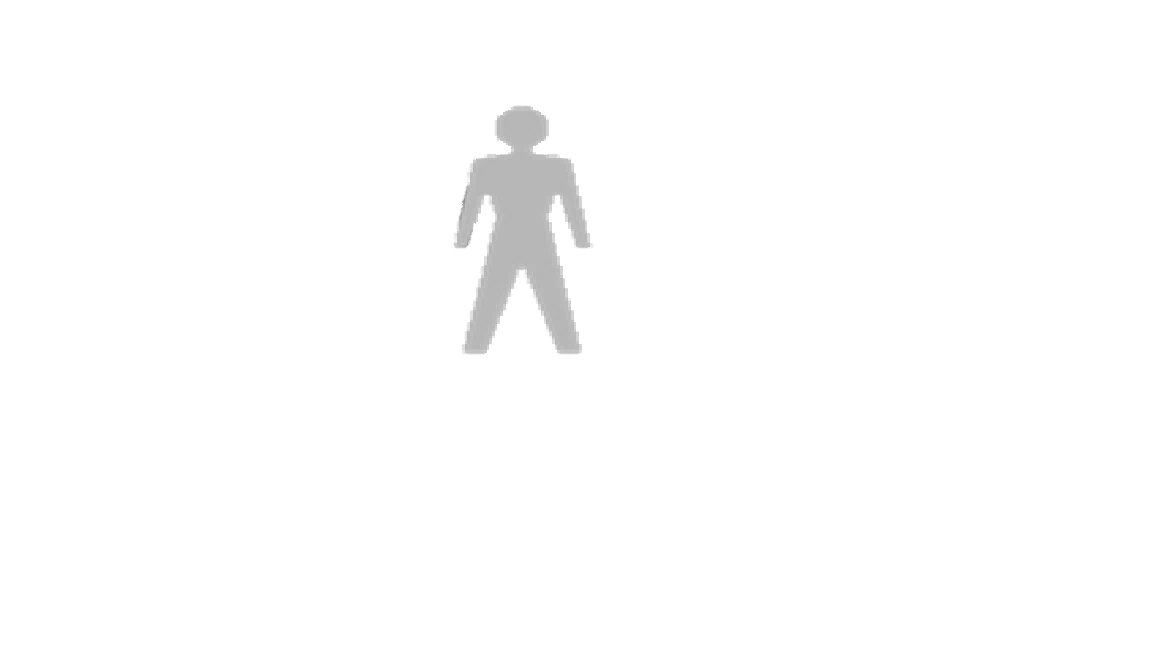

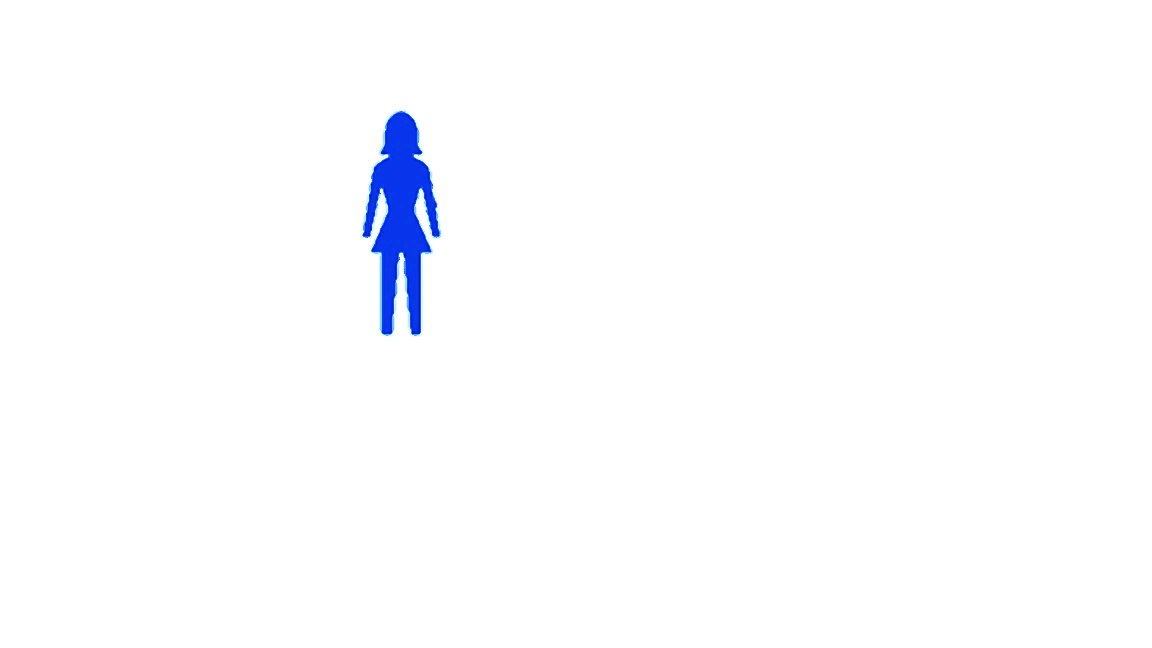

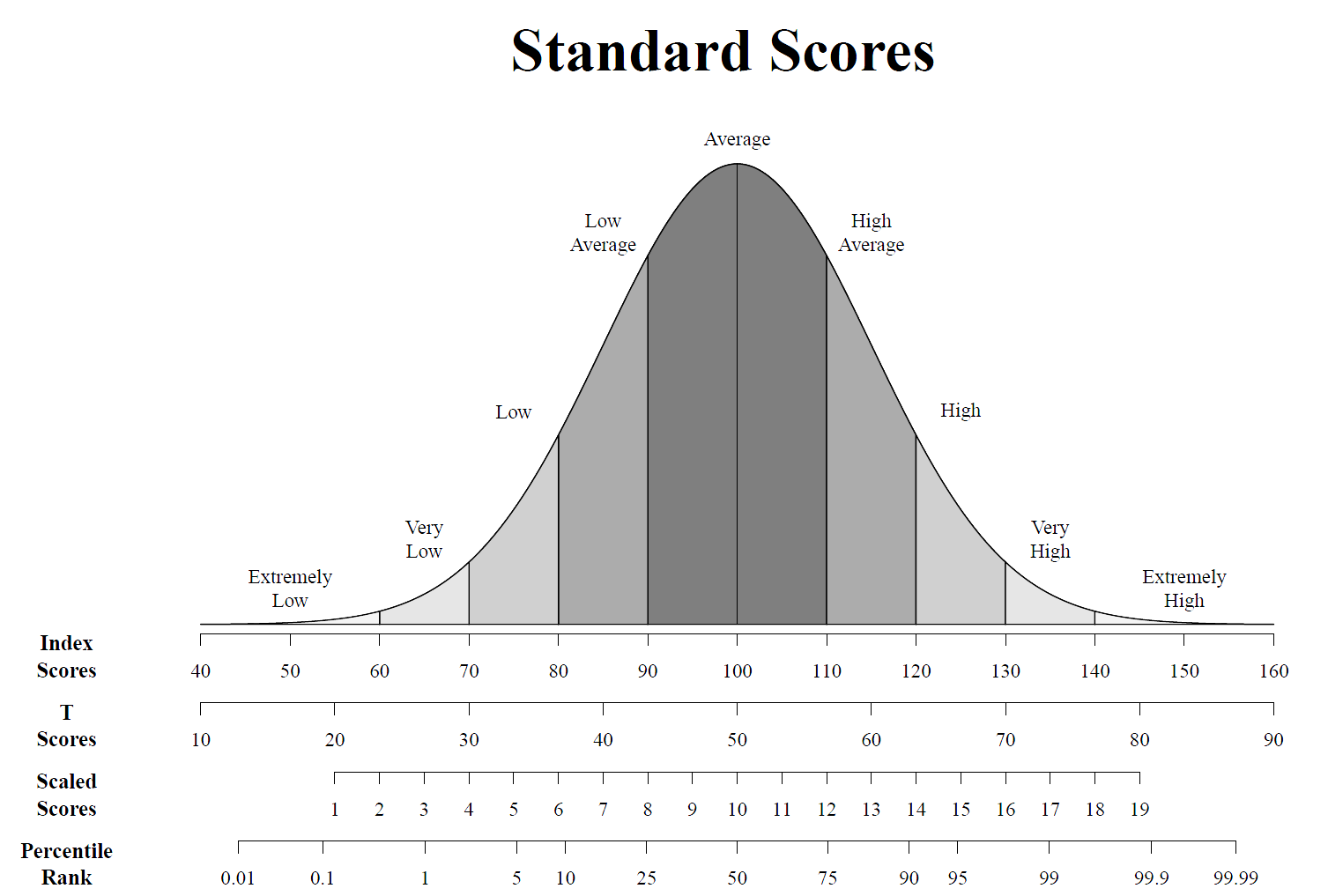


**Person 2**

**Rank 14**

**Person 1**

**Rank 81**

**The Average The Worst Best**
